# An Evaluation of the Swissmedic Regulatory Framework for New Active Substances

**DOI:** 10.1007/s43441-023-00581-7

**Published:** 2023-10-26

**Authors:** Magda Bujar, Simon Andreas Dalla Torre di Sanguinetto, Adem Kermad, Claus Bolte, Neil McAuslane

**Affiliations:** 1 Centre for Innovation in Regulatory Science, London, UK; 2 Swissmedic, Swiss Agency for Therapeutic Products, Bern, Switzerland

**Keywords:** Regulatory, Performance, Metrics, Opera, Swissmedic

## Abstract

**Background:**

Swissmedic is a major regulatory agency that has been benchmarking its timelines for 20 years. To better understand the Swissmedic review times and to examine whether measures introduced to accelerate the process were effective, a retrospective analysis was undertaken. The objective was to provide a breakdown of where time is spent in the phases of Swissmedic’s approval process (validation, scientific assessment, authorisation) and how this compared to other major authorities.

**Methods:**

Data on Swissmedic, EMA and FDA product approvals were collected from websites or through direct communication, using a standardised CIRS method and milestones previously identified, focusing on new active substances approved 2019–2021.

**Results:**

In 2019, 2020, and 2021, Swissmedic median approval times were 520, 470, and 392 days, respectively. The decrease over this time was mainly observed in the Authorisation Phase and can be attributed to lower proportions of applications with multiple “labelling loops”, in addition to shorter times for final label negotiation. While Swissmedic had the longest overall approval time (447 days) compared to EMA (428) and FDA (244), the timelines were more comparable when considering only the agency’s time spent on the scientific assessment, with Swissmedic at 194 days, EMA at 218 days, and FDA at 184 days.

**Conclusions:**

These observations represent an important analysis of Swissmedic regulatory activity timelines, demonstrate the impact of process improvements, and emphasise the importance of measuring timelines. Swissmedic will continue to expedite its processes also by promoting international collaborations with like-minded authorities.

## Introduction

### Evaluating the Regulatory Process

Regulatory approval time is a key metric that has been used to assess and compare the performance of regulatory authorities globally. As the regulatory processes of agencies evolve, documenting performance against review target timelines may help to assess the process limitations and identify the areas for improvement. This provides organisations with insights into regulatory processes and practices, with a goal of ensuring an efficient and effective process and ultimately enabling timely availability of medicines.

Recognising the importance of advancing regulatory practices, Centre for Innovation in Regulatory Science (CIRS) has been benchmarking major regulatory agencies since 2002 using a methodology developed with the authorities, including Swissmedic [1], to provide insights into regulatory processes, identify where improvements can be made, and inform company and agency strategies. The study now focuses on the review of new active substances (NASs) by six regulatory agencies: the U.S. FDA, EMA, Japanese PMDA, Health Canada, Swissmedic and Australian TGA [2]. Building on this original work, more recently, the CIRS “Optimising Efficiencies in Regulatory Agencies” (OpERA) programme was initiated by CIRS in 2013 to similarly benchmark and strengthen agencies from Asia, Latin America, Africa, and the Middle East and is available to all regulatory agencies irrespective of their size, mission, or maturity [3].

The agencies participating in the OpERA programme have identified commonly collected milestones, e.g. receipt of the dossier, start, and end of scientific assessment as well as time to send deficiency questions and/or the industry response time to those queries, which can be used to demonstrate both the total agency and company time associated with the medicine review process [3]. Results obtained from CIRS benchmarking analyses can therefore help agencies identify where time is spent in their processes, define their regulatory performance goals, monitor change activities, embed a culture of ongoing self-assessment, optimise their process efficiencies, and increase transparency.

### Optimisation of the Swissmedic Review Process

**Figure 1:**
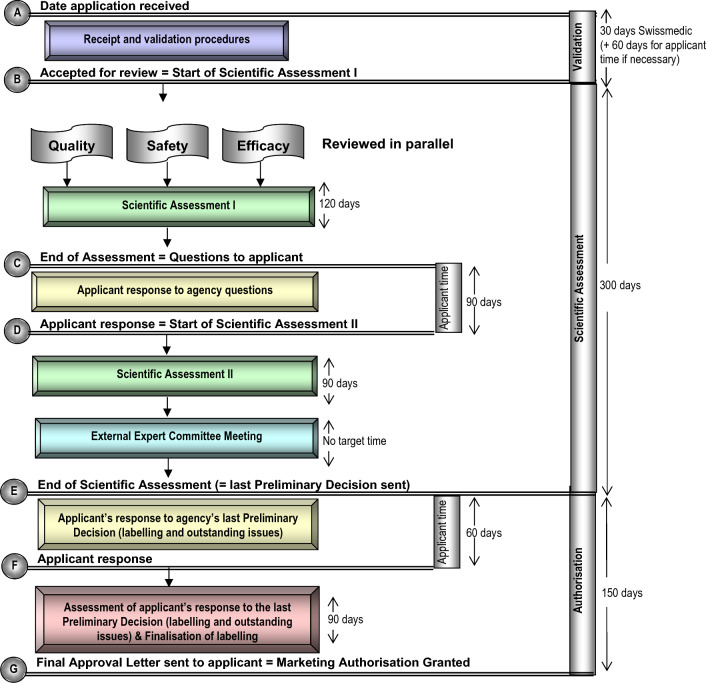
Swissmedic process map (calendar days).

Within the framework of the authorisation procedure, Swissmedic assesses the quality, safety, and effectiveness of the medicinal product in question on the basis of the comprehensive scientific documentation that is submitted. For the purposes of this study, the term "standard review process" is used for the authorisation of new medicinal products when no additional criteria for a facilitated or accelerated authorisation pathway are met. The Swissmedic standard review process is described in Fig. 1, where the OpERA methodology was utilised to map out the Swissmedic process for the authorisation of new medicines, using the common OpERA milestones. In the standard review process, the overall target assessment time from the submission date to the final decision is 330 calendar days for Swissmedic and 210 calendar days for the applicant. These timelines include the validation of the application dossier and the scientific assessment by Swissmedic as well as the applicant’s response to the Swissmedic list of questions as well as the Authorisation Phase where of the drug information texts are finalised in consultation with the applicants.

In international comparisons of the processing time for the scientific assessment only, Swissmedic was shown to be comparable with the fastest authorities undertaking a full review on new medicines [2, 4–6]. However, the total Swissmedic approval time was considerably longer than those of the fastest agencies. In contrast to other authorities, Swissmedic spends a relatively large amount of time outside the actual scientific assessment, particularly in the Authorisation Phase. This final phase follows the Scientific Assessment Phase and serves to finalise the summary of product characteristics (SmPC) in consultation with the applicant (therefore referred to as Labelling Phase at Swissmedic). Although not officially foreseen in the standard review process, this Swissmedic Labelling Phase may occur through multiple negotiation “loops” between the industry and the agency (hereafter called “labelling loops”). Thus, in response to the findings on the loss of time outside of scientific assessment, between October 2017 and July 2020 Swissmedic has taken a number of targeted measures, particularly with the goal to reduce the number of additional labelling loops and to thereby shorten the Labelling Phase:

1. Earlier review of the summary of product characteristics and package elements
  With this measure, Swissmedic switched from assessing the SmPC and the submitted package elements during Scientific Assessment Phase I instead of Phase II. According to the new process, Swissmedic uses the List of Questions to provide first feedback to the applicant on which elements need to be corrected so that a consolidated version of the SmPC and the package elements is available at the time of the Preliminary Decision.
2. Exercise of official authority
  In the past, the completion of the application was sometimes protracted due to labelling negotiations without the applicant presenting new facts to support its position. With the implementation of this measure, Swissmedic decrees the drug information texts without further involvement of the applicant.
3. Shortening of company deadlines in the Labelling Phase
  In the past, applicants were given a standard deadline of 90 calendar days to respond to the Preliminary Decision. With this measure, this period has been shortened to 60 calendar days. Similarly, the deadline for responding to an additional labelling loop has been reduced from 90 to 30 calendar days.
4. Intensified dialogue with the applicant in the Labelling Phase
  With this measure, Swissmedic has established a new procedure to clarify the questions regarding labelling with the applicant with very short deadlines, thereby avoiding additional formal labelling loops.

In order to better understand the Swissmedic timelines and in order to verify whether the measures described to accelerate the Labelling Phase were effective, a retrospective analysis of the timelines associated with important milestones of the review process (addressing both agency and company time) was required. The objective of this study was therefore to provide Swissmedic with a breakdown of where time is spent in their approval process, and how this compared to other major regulatory authorities. The main goal was not only to assess agency practices and identify further areas for improvement, but also to form a baseline against which future performance improvements can be measured.

## Methods

### Data Collection

Swissmedic provided to CIRS information on product characteristics and regulatory milestone dates consistent with those collected through the OpERA programme [3]. Data were provided by Swissmedic in a spreadsheet contain individual pharmaceutical product applications and their corresponding dates for the milestones described in Table 1. Unless otherwise specified, all times are indicated in calendar days (hereafter “days”). The collected data enabled the calculation of time for the following key phases:

- *Validation*: The time between the date stamped on receipt of the dossier and the beginning of the Scientific Assessment Phase I.
- *Scientific assessment*: The time spent between the date of the start of the scientific assessment and the date of completion of all scientific assessments. This constitutes:
  - Agency scientific assessment time I and II.
  - Applicant time to respond to Swissmedic list of questions and/or initial Preliminary Decision in case a second Preliminary Decision must be issued.
- *Authorisation*: The time from completion of all the scientific assessment to the official decision date of legal marketing.
  - At Swissmedic, the Authorisation Phase includes the negotiation time with the applicant regarding the label following the end of the scientific assessment (= date when the last preliminary decision is sent). Although not foreseen in the standard review process, this Swissmedic Labelling Phase may occur through multiple negotiation “loops” between the industry and the agency.
- *Total approval*: The time between the date stamped on the receipt of dossier when received by authority and the date on the official decision letter that allows legal marketing.

**Table 1:** Phases, Milestones, and Intervals Used for Calculating the Swissmedic Timelines as Mapped in Fig. 1

Phase	Type	Swissmedic milestones	Process map interval (Fig. 1)
Validation	Agency and company time	Submission date—accepted for review	A → B
Scientific assessment			
Scientific assessment I	Agency time only	Accepted for review—list of questions (LoQ) or preliminary decision sent to applicant (if no LoQ is issued)	B → C
Applicant response to agency questions	Company time only	LoQ sent to applicant—applicant’s answers to LoQ received	C → D
Scientific Assessment II	Agency time only	Applicant’s answers to LoQ received—preliminary decision sent to applicant	D → E
Authorisation (Swissmedic Labelling Phase)			
Applicant’s response to agency’s last Preliminary Decision	Company time only	Last preliminary decision sent to applicant—applicant’s answers to the last preliminary decision received	E → F
Assessment of applicant’s response to the last Preliminary Decision	Agency (and company time in case of additional “labelling loops”)	Applicant’s answers to preliminary decision received—official approval letter sent to applicant (granting of marketing authorisation)	F → G
Total approval	Agency and company time	Submission date—official approval letter sent to applicant (granting of marketing authorisation)	A → G
Total agency scientific assessment	Agency time only	Scientific assessment I + scientific assessment II	(B → C) + (D → E)
Total agency and company scientific assessment	Agency and company time	Start of scientific assessment I—preliminary decision sent to applicant	B → E

CIRS evaluated and validated the integrity of the information provided. A draft analysis was prepared and presented in a virtual meeting with Swissmedic to ensure agency understanding of the dataset and consistency of data provision. The data for EMA and FDA, as well as the corresponding target assessment timelines, were collected from the agencies’ websites (Table 2).

**Table 2:** Target Timelines for Swissmedic, EMA, and FDA Standard Review Processes for New Active Substances

Phase	Validation	Scientific assessment	Authorisation
Swissmedic	30 days + 60 days for applicant in case of an incomplete dossier	Agency—210 days total	60 days for applicant to respond to Preliminary Decision
		120 days for scientific assessment I	
		90 days for scientific assessment II	90 days for agency to respond to applicant’s response on preliminary decision
		Applicant: 90 days for applicant response to LoQ	
EMA	13 working days + ~ 2 months for applicant reply	Agency (210 days total):	67 days for European Commission
		120 days for scientific assessment I	
		90 days for scientific assessment II	
		Applicant: 2 months clock stop after Sci. Assessment I can be extended up to 6 months	
FDA	60 days for filing notification to applicant	Agency: 10 months first cycle review (scientific assessment I)	Not applicable [the FDA action letter to approve is signed (FDA action date)]. This is equivalent to the regulatory approval, and therefore for FDA, time from acceptance of submission to end scientific assessment and time from acceptance of submission to approval are the same
		Up to 6 months for resubmission evaluation (scientific assessment II)	
		Applicant: 30 days to respond to complete response letter and request extension	

The following metrics were collected in addition to the milestones described in Tables 1 and 2: product name; applicant name; company; compound type; ATC code (as defined by the WHO); use of work-sharing and collaborative reviews via Access Consortium (https://www.swissmedic.ch/swissmedic/en/home/about-us/international-collaboration/multilateral-co-operation-with-international-organisations---ini/multilateral-co-operation-with-international-organisations---ini.html) and Project Orbis (https://www.fda.gov/about-fda/oncology-center-excellence/project-orbis), respectively; review type: standard review, fast-track authorisation procedure (FTP) [7]; or the Procedure with Prior notification (PPN) [8]. Other authorisation pathways, such as the conditional/time-limited marketing authorisations, exist at Swissmedic as well as in other jurisdictions, but were not investigated in this study. The target timelines for the examined authorisation pathways were retrieved from the Swissmedic Guidance document *Time limits for authorization applications.*

[Reference]: https://www.swissmedic.ch/dam/swissmedic/en/dokumente/zulassung/zl_hmv_iv/zl000_00_014d_wlfristenzulassungsgesuche.pdf.download.pdf/zl000_00_014e_wltimelimitsforauthorizationapplications.pdf.

### Scope

This analysis focused on new active substances (NAS) applications approved between 1st January 2019 and 31st December 2021 by Swissmedic, EMA (according to the European Commission date) or FDA.

NAS was defined by CIRS as follows:

- A chemical, biological, biotechnology, or radiopharmaceutical substance that has not been previously available for therapeutic use in humans and is destined to be made available as a ‘prescription-only medicine’, to be used for the cure, alleviation, treatment, prevention, or in vivo diagnosis of diseases in humans.
- An isomer, mixture of isomers, a complex or derivative or salt of a chemical substance previously available as a medicinal product but differing in properties with regard to safety and efficacy from that substance previously available.
- A biological or biotech substance previously available as a medicinal product, but differing in molecular structure through changes to the nature of source material or manufacturing process and requiring clinical investigation.
- A radiopharmaceutical substance that is a radionuclide or a ligand not previously available as a medicinal product—alternatively, the coupling mechanism linking the molecule and the radionuclide has not been previously available.

The following entities were excluded:

- Vaccines,
- Biosimilars,
- Any other application, where new clinical data were submitted,
- Generic applications,
- Those applications where a completely new dossier was submitted from a new company for the same indications as already approved for another company,
- Applications for a new or additional name, or a change of name, for an existing compound, that is, a ‘cloned’ application.

As a result of using this definition, the NAS numbers may differ from the approval numbers published by the respective agencies.

### Analysis

Unless otherwise specified, all times are indicated in calendar days (hereafter “days”). Data were described statistically using medians and percentiles, in particular, the 25th and 75th percentiles, to facilitate the understanding of the variation around the median (50th percentile).

## Results

The results are presented in three parts:

- Part I: Overall Swissmedic process
- Part II: Swissmedic process milestones
- Part III: International Comparison

### Part I: Overall Swissmedic Process

**Figure 2:**
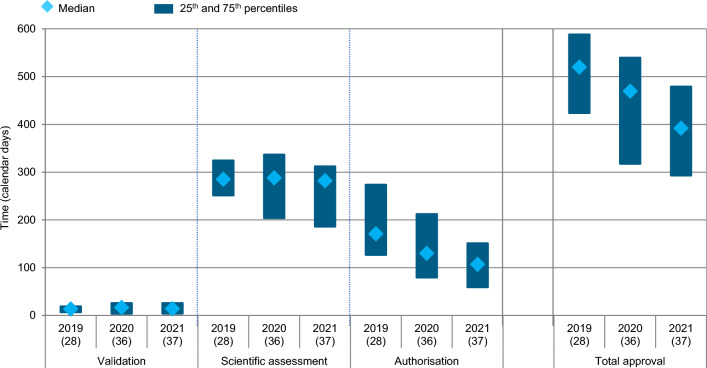
Swissmedic regulatory review times for all NASs (all regulatory pathways considered)) approved 2019–2021 (*n*) = number of NAS applications.

The total median approval time (including company and agency time) for all NASs approved by Swissmedic were 520, 470, and 392 days in 2019, 2020, and 2021, respectively, with 28 NAS approvals in 2019, 36 in 2020, and 37 in 2021. The timelines broken down according to the three major phases (1) validation, (2) scientific assessment, and (3) authorisation (referred to as the Labelling Phase by Swissmedic) were analysed by year of approval (Fig. 2). For validation, the median times were 17 days in 2019–2021 and were consistent across the years also regarding the variance around the median. The median scientific assessment time for 2019–2021 was 286 days. For the individual years, median scientific assessment time was relatively consistent (285, 288, and 282 days for 2019, 2020, and 2021, respectively), albeit with greater variance between the 25th and 50th percentiles in both 2020 and 2021. Finally, the median authorisation time was 135 days in 2019–2021, with a year-to-year decrease from 171 days in 2019 to 107 days in 2021.

For FTP, target agency review times are shortened by 58% compared to standard review (140 days vs. 330 days) and company response time is the same as standard review process, resulting in a total target time reduction of 35%. For PPN, target agency review times are 20% shorter compared to standard review process (266 vs. 330 days); and company response times are 24% (160 days vs. 210 days) shorter, resulting in a total target time reduction of 21% [8].

**Figure 3:**
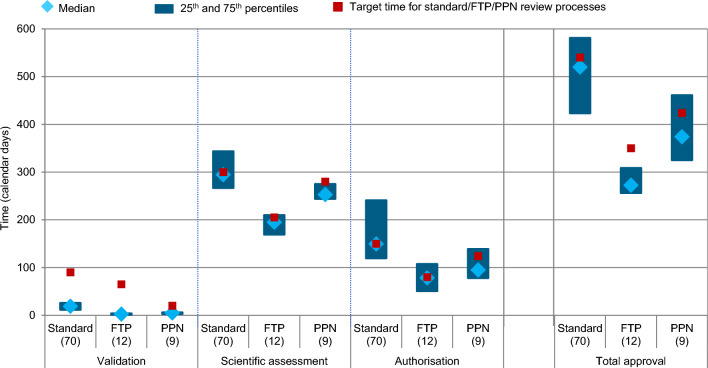
Swissmedic regulatory review times for NASs approved 2019–2021 broken down by the type of review: standard review process (non-FTP, non-PPN; Orbis, and Access products excluded), FTP and PPN according to the main phases of the marketing authorisation process. (*n*) = number of NAS applications.

The proportion of Swissmedic FTP was 7% (2 out of 28), 19% (7 out of 36), and 8% (3 out of 37) in 2019, 2020, and 2021, respectively. The proportion of NASs reviewed through the PPN comprised 14% (4 out of 28), 11% (4 out of 36), and 3% (1 out of 37) of the NAS approvals during those years. In 2019–2021, FTP resulted in the fastest validation, scientific assessment, as well as authorisation times with 2.5 days, 195 days, and 78.5 days median, respectively, compared to standard review process, where the three phases had medians of 19, 294.5, and 149.5 days, respectively. Most notably, the median scientific assessment time was 99.5 days faster for FTP compared to the standard review process, and the median authorisation time was 71 days faster. Similarly, NAS approved through the PPN was considerably faster than the standard review applications in all three phases with 5, 253, and 95 days, respectively (Fig. 3). In addition, the accelerated authorisation pathways FTP and PNN were also generally more predictable as noted by smaller variance around the median.

In 2019–2021, four NASs were approved through the Access Consortium (1 in 2020; 3 in 2021) and the median approval time for those products was 376 days. Finally, six NASs were approved through Project Orbis (1 in 2020; 5 in 2021), where the median approval time was 275 days. Due to the limited number of applications, these results were not presented graphically.

### Part II: Swissmedic Process Milestones

**Figure 4:**
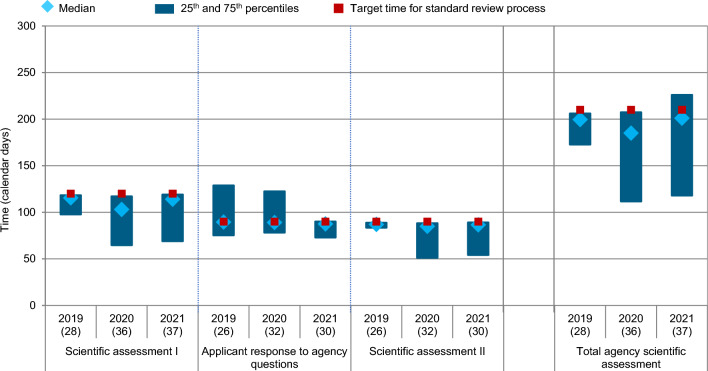
Swissmedic regulatory review time for all NASs (all regulatory pathways considered) approved 2019–2021 (*n*) = number of NAS applications.

The timeline for scientific assessment was further broken down according to the individual milestones within the process—primary scientific assessment, company response time during scientific assessment, as well as secondary scientific assessment (Fig. 4). The primary and secondary scientific assessment times were combined to calculate the total agency scientific assessment time with a median timeline of 200, 185, and 201 days, respectively, in 2019, 2020, and 2021. All median times, within each process interval but also for each approval year, were within the target timelines for standard review based on the guidelines stipulated by the agency.

**Figure 5:**
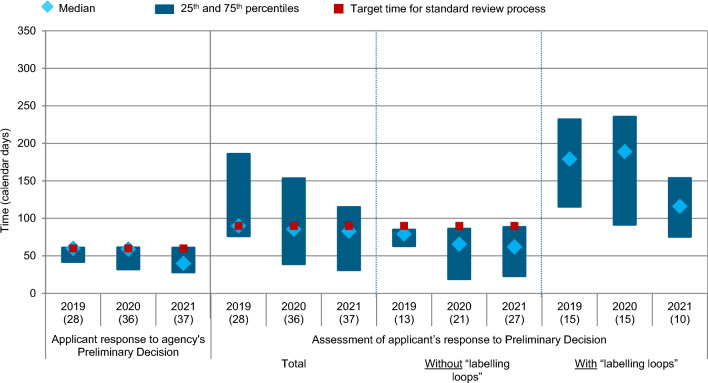
Swissmedic regulatory review time for all NASs (all regulatory pathways considered) approved 2019–2021—breakdown of the Authorisation Phase compared to the agency target timeline. (*n*) = number of NAS applications.

In order to develop a better understanding of how Swissmedic and the applicants use their time in the labelling negotiations of the Authorisation Phase, the phase was further broken down into its components (Fig. 5). However, it should be noted that all regulatory pathways, including the standard review process, FTP, and PPN, as well as the international pathways Orbis and Access were included in the analysis for Figs. 4 and 5. For ease of readability, only the target time benchmarks for the standard review process were shown as red boxes. As to be expected, the median review times for all regulatory pathways are considerably lower than the target times of the standard review process.

The applicants’ median time to respond to the agency’s Preliminary Decision was largely within the 60 days target timeline. Similarly, Swissmedic generally reviewed the applicants’ responses with the foreseen 90 days (Fig. 5, referred to as “Total”). For the subgroup of applications for which Swissmedic can issue a final decision directly after assessing the applicants’ responses to the Preliminary Decision (referred to as “Without labelling loops”), the Swissmedic time was fast: the 75th percentile which was within the 90 days target and the median decreased slightly over the years with 79, 66, and 62 days in 2019, 2020, and 2021, respectively. For the subgroup of applications for which further queries to the applicant were necessary (referred to as “With labelling loops”), the process to issue a final decision took considerably longer and in addition to agency time (target time 90 days) also comprised applicants time required to answer Swissmedic’s questions (target time 30 days). Please note that the proportion of applications with the time-consuming multiple “labelling loops” decreased steadily from 54% in 2019, compared to 42% in 2020 and 27% in 2021, respectively. At the same time, when additional “labelling loops” still occurred, the time needed to reach the milestone of the official decision also decreased from 179 days in 2019 to 116 days in 2021.

### Part III: International Comparison

**Figure 6:**
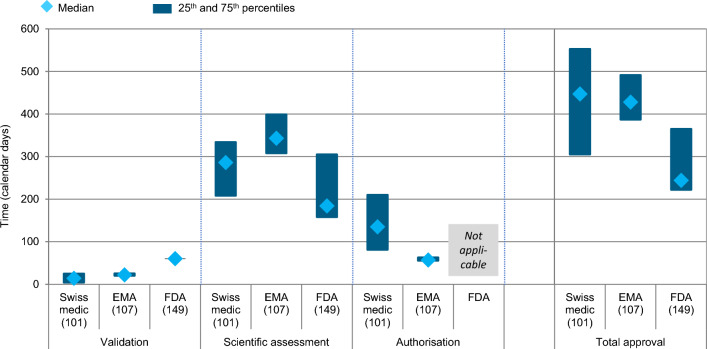
Swissmedic regulatory review times for all NASs (all regulatory pathways considered) approved 2019–2021—compared to EMA and FDA. (*n*) = number of NAS applications.

The Swissmedic approval times were compared to those of EMA and FDA (Fig. 6). For 2019–2021, the total median approval times for Swissmedic were the longest, with 447 days compared to EMA and FDA with median times of 428 and 244 days, respectively, and displayed the biggest variance compared to the other two agencies. When comparing validation, FDA times were longest; followed by EMA and Swissmedic. However, the opposite was the case for scientific assessment where FDA had the shortest median times, followed by Swissmedic and lastly EMA. For FDA, the end of scientific assessment is equivalent to marketing authorisation (i.e. there is no additional Authorisation Phase). For Europe, the median Authorisation Phase, which is the European Commission time, took 57 days, compared to Swissmedic with 135 days.

**Figure 7:**
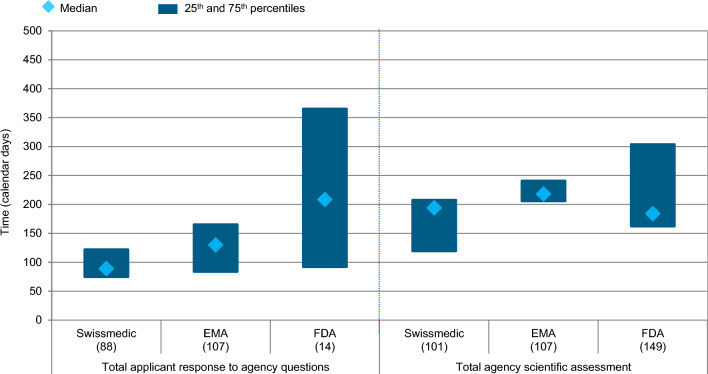
Swissmedic regulatory review times for all NASs (all regulatory pathways considered) approved 2019–2021 according to total applicant and agency time during scientific assessment—compared to EMA and FDA. (*n*) = number of NAS applications.

Lastly, the Swissmedic total agency scientific assessment time as well as applicant time following scientific assessment was compared to EMA and FDA (Fig. 7). In 2019–2021, the total agency timelines were comparable for the three agencies with 184 days for FDA, followed by 194 days for Swissmedic and 218 days for EMA. Scientific assessment occurred in multiple cycles for all EMA products (107), 88 out of 101 Swissmedic products and 14 out of 149 products for FDA (i.e. where complete response letter was received). For multiple cycle applications, the applicant time was calculated. It was the longest for FDA with a median of 209 days compared to 130 days for EMA and 89 days for Swissmedic.

## Discussion

Swissmedic is a major regulatory agency that not only regulates medicinal products in Switzerland, but also participates in global activities with other like-minded regulators, which enables the agency to be highly efficient and expedite the approval of medicines. The primary reason for this study was to provide an understanding of where time is being spent within the Swissmedic review process and to verify whether the measures described to accelerate the Labelling Phase were effective. This is the only recent study that evaluates both agency and company times within the Swissmedic review process, also in comparison with other regulators, based on data collected primarily from the public domain where previous studies focused on overall approval times only or data only collected directly from agencies [1, 2, 6, 9, 10].

### Improvements in Swissmedic Review Process

The findings represent an important analysis of the breakdown of the Swissmedic regulatory times, across multiple years and review types. Overall, the results demonstrate that the regulatory review times, based on the median, are meeting the target timelines stipulated by the agency. In addition, across the 3 years 2019–2021, the median review times have decreased steadily, despite the challenges such as the COVID-19 pandemic, suggesting that the measures that the agency has put in place have had a positive impact in accelerating the regulatory review process.

The rational for the decrease in the overall review times was studied by analysing each phase of the process: Validation, Scientific Assessment, and Authorisation. The results demonstrated that the validation time remained consistent and in line with the Swissmedic target timeline over 2019–2021. Similarly, the overall scientific assessment time remained consistent in terms of the median; however, it slightly decreased when comparing the 25th percentiles (Fig. 4). This may be due to an efficient use of FTP, PPN, as well as the recently introduced collaborative international pathways Access and Orbis. Taken together, the proportion of these accelerated regulatory pathways increased from 21% in 2019, over 36% in 2020 to 32% in 2021, strongly driven by the increasing use of Access and Orbis pathways.

Of the three examined phases, the largest decrease in the median time from 2019 to 2021 is in the Authorisation Phase. A further breakdown of the Authorisation Phase showed that the applicant time to respond to the last Preliminary Decision was relatively consistent over 2019–2021 and in line with Swissmedic target timelines. In contrast, the efficiency of the authorisation process appeared to improve from 2019 to 2021, through both a decrease in the proportion of applications that have multiple “labelling loops” and a decrease in the time needed to negotiate the final label and issue the marketing authorisation decision. The authors identify these effects as the strongest driver regarding the overall reduction of approval times (Fig. 2) and they were likely the result of the four Swissmedic measures particularly targeted at shortening the Authorisation Phase as described above. Although it was not the aim of the present study to examine in detail the influence of the individual measures on reducing the approval times, Swissmedic assumes that the greatest influence was achieved by frontloading the review of the SmPC. With this particular measure, applicants received initial feedback on the SmPC already with the List of Questions. Therefore, at the time of the Preliminary Decision, a more complete version of the SmPC is available and the probability that additional labelling loops will be necessary for the finalisation of the SmPC is reduced.

### International Comparison

A comparison of Swissmedic with other major regulators, EMA and FDA, highlighted that although the three agency’s total approval time are comparable, there are notable differences in how the three agencies review medicines based on their processes and legislations. For example, FDA has a longer Validation Phase prior to the scientific assessment where the scientific context of the dossier is already being reviewed and the quality and completeness of the dossier is also verified. In comparison, at the EMA, the validation process comprises more administrative activities using checklists to ensure the completeness of the documents. In addition, Swissmedic's (as well as EMA’s) scientific assessment is usually done in two main steps (Scientific Assessment I and II), while at the FDA, most applications are reviewed in just one cycle and information requests are made and answered throughout the review process. At the end of the scientific assessment, the FDA may issue "complete response" letters if approval is not possible. While the application remains open, the applicant is given time to present additional scientific evidence in support of the application. This explains FDA's considerably longer "applicant response time to agency questions". Finally, only EMA and Swissmedic have the additional Authorisation Phase following the end of scientific assessment, where for EMA this is the European Commission step and for Swissmedic this phase focuses on labelling negotiations. Overall, the comparative assessment has shown that a like-with-like comparison of agency regulatory review times can be undertaken more effectively by comparing the individual components of the review, e.g. validation, scientific assessment, and authorisation but also by analysing the total agency time during scientific assessment. Consequently, the detailed OpERA method is more constructive compared to just analysing the overall regulatory review time, as it highlights where time is spent and where additional improvements can be made in order to ensure an even more effective and efficient process.

### Swissmedic Way Forward

As all drug regulatory authorities, Swissmedic aims to make safe and effective innovative medicinal products available to patients as quickly as possible by means of a rapid and efficient assessment. The annual benchmarking of authorisation times (Ref. CIRS R&D Briefing 85, 88) shows how well Swissmedic fulfils this central task compared to other international authorities. As the results show, Swissmedic has been able to accelerate its approval times by more than 120 days between 2019 and 2021. Among other, Swissmedic’s measures targeted to avoid additional labelling loops in the Authorisation Phase may have contributed to this acceleration. Despite this favourable development, Swissmedic is continuing its efforts to further optimise the Authorisation Phase, also in collaboration with external stakeholders. However, Swiss procedural law requires a two-stage decision-making process with a hearing right for the applicant, followed by the final decision. Due to this regulation, certain delays after completion of the scientific assessment cannot be completely avoided in the future.

Besides Swissmedic’s efforts to optimise its assessment processes, international collaborations have equally contributed to the overall acceleration of authorisation times. Firstly, since 2020, Swissmedic has been participating in the FDA's Orbis project. The FDA Oncology Center of Excellence initiated Project Orbis in May 2019 to provide a framework for concurrent submission and review of oncology products among international partners with the aim to allow patients with cancer to receive earlier access to innovative medicinal products (https://www.fda.gov/about-fda/oncology-center-excellence/project-orbis). Swissmedic not only benefits from the scientific discussions with the FDA and the other participating regulatory authorities, but approvals under Project Orbis are characterised by short submission gaps and approval times (CIRS R&D Briefing 85, 88).

Secondly, Swissmedic has been part of the Access Consortium since 2019 (REF to Swissmedic Access page: https://www.swissmedic.ch/swissmedic/en/home/about-us/international-collaboration/multilateral-co-operation-with-international-organisations---ini/multilateral-co-operation-with-international-organisations---ini.html).

The Access Consortium is a collaborative initiative of like-minded, medium-sized regulatory authorities between Australia's Therapeutic Goods Administration (TGA), Health Canada (HC), Singapore's Health Sciences Authority (HSA), and the Medicines and Healthcare products Regulatory Agency (MHRA) of the United Kingdom and Swissmedic. The purpose of the Consortium is to build synergies and share knowledge among the regulatory authorities thereby enhanced efficiency of regulatory systems facilitates work-sharing on medicines and reduces duplication of work. In terms of approval times, the Access work-sharing pathway is also characterised by shortened review timelines, approval times, and thus accelerated availability of medicines to patients (Ref. CIRS R&D Briefing 85, 88).

Although only a limited number of products reviewed as part of the Access Consortium or Project Orbis were captured through this analysis, some positive impact on the overall approval times was shown. As the number of products approved through these collaborative international pathways is increasing, it should be monitored whether a further acceleration of the authorisation processes can be achieved at Swissmedic.

Swissmedic will continue to promote and foster cooperation within the framework of the Access Consortium and Project Orbis as well as other international collaborations with like-minded authorities. Swissmedic is convinced that strategic international collaboration not only has the potential to bring medicines to patients more quickly, but also serves to stay scientifically state-of-the art and to save resources through work-sharing.

Following several years of continued process improvement initiatives in close collaboration with industry stakeholders and international regulatory partner agencies, Swissmedic has reached a point where market access ought to be looked at in a more holistic manner. Hence, health-economic data should be submitted to the health technology assessment (HTA) agency in parallel with the regulatory dossier to Swissmedic. Ideally, evidentiary requirements for reimbursement and (value-based) pricing will be discussed at joint regulatory & HTA meetings already in development. In a best-case scenario, regulatory and reimbursement processes will be increasingly aligned and synchronised, starting early in development and including scientific advice as well as HTA advice, enabling fast market access for innovative pharmaceutical products with added value, including reimbursement. [Reference].

[Reference]: “Innovation and Market Access”—Bolte, C; in ‘Die Schweiz 2030—Switzerland 2030’ published by the Swiss Federal Administration, 17.10.2018 NZZ Libro, ISBN 978-3-03810-360-8. http://www.nzz-libro.ch/schweiz-suisse-svizzera-2030-bundeskanzlei-buch.html.

### Impact and Future OpERA Research

CIRS has been benchmarking regulatory authorities globally to support efficient, effective, and fit for purpose regulatory systems. This publication forms part of CIRS OpERA work to optimise efficiencies in regulatory authorities [7, 11]. The breakdown of company and agency time as well as international comparisons demonstrate the benefits of such as study to understand where time is spent in regulatory review process and how that differs across regulators, as well as the ability to demonstrate the impact of measures introduced by agencies to improve their process. Agencies are seeing the benefit of such analyses to obtain factual, independent information that allows for the identification of improvements that are resource dependent in order to make a stronger case for the additional resources. In addition, a common methodology developed with agencies results in data that is comparative across other agencies.

There is a growing interest from stakeholders to understand Swissmedic timelines, e.g. from other agencies looking to workshare/partner with Swissmedic or utilise it as a reference agency. Future studies may therefore focus on comparing Swissmedic to other regulators, those part of the Access Consortium or Orbis Worksharing which Swissmedic participates in, as well as other mid-size regulators globally that are similarly resourced. Here, the OpERA methodology could again be utilised to ensure like-with-like comparisons of agency review times based on the common milestones.

## Conclusion

These observations represent an important analysis of Swissmedic regulatory activity timelines across multiple years and review types and demonstrate the impact of process improvements on ensuring timely approval of medicines in Switzerland. Future research will focus on assessing the impact of increasingly important strategic partnerships such as Access and Orbis as further measures to ensure regulatory efficiency and effectiveness.

## Data Availability

Parts of the raw data used to evaltuate Swissmedic's processes are not in the public domain. The compounds included in this study are available on CIRS website e.g. https://cirsci.org/wp-content/uploads/dlm_uploads/2022/06/CIRS-RD-Briefing-85-NAS-list-v2.2-reuploaded.pdf, https://cirsci.org/wp-content/uploads/dlm_uploads/2021/06/CIRS-RD-Briefing-81-NAS-list-v3.pdf.
